# Potential prognostic impact of EBV RNA‐seq reads in gastric cancer: a reanalysis of The Cancer Genome Atlas cohort

**DOI:** 10.1002/2211-5463.12803

**Published:** 2020-02-16

**Authors:** Daichi Sadato, Mina Ogawa, Chizuko Hirama, Tsunekazu Hishima, Shin‐Ichiro Horiguchi, Yuka Harada, Tatsu Shimoyama, Masanari Itokawa, Kazuteru Ohashi, Keisuke Oboki

**Affiliations:** ^1^ Division of Hematology Tokyo Metropolitan Cancer and Infectious Diseases Center Komagome Hospital Bunkyo‐ku Japan; ^2^ Center for Medical Research Cooperation Tokyo Metropolitan Institute of Medical Science Setagaya‐ku Japan; ^3^ Divisions of Clinical Research Support Tokyo Metropolitan Cancer and Infectious Diseases Center Komagome Hospital Bunkyo-ku Japan; ^4^ Department of Medical Oncology Tokyo Metropolitan Cancer and Infectious Diseases Center Komagome Hospital Bunkyo‐ku Japan; ^5^ Department of Pathology Tokyo Metropolitan Cancer and Infectious Diseases Center Komagome Hospital Bunkyo‐ku Japan

**Keywords:** bioinformatics, EBV, gastric cancer, histopathological subtype, RNA‐seq, survival analysis

## Abstract

Epstein–Barr virus (EBV)‐associated gastric cancer (EBVaGC), whose prognosis remains controversial, is diagnosed by *in situ* hybridization of EBV‐derived EBER1/2 small RNAs. In The Cancer Genome Atlas (TCGA) Stomach Adenocarcinoma (STAD) project, the EBV molecular subtype was determined through a combination of multiple next‐generation sequencing methods, but not by the gold standard *in situ* hybridization method. This leaves unanswered questions regarding the discordance of EBV positivity detected by different approaches and the threshold of sequencing reads. Therefore, we reanalyzed the TCGA‐STAD RNA sequencing (RNA‐seq) dataset including 375 tumor and 32 normal samples, using our analysis pipeline. We defined a reliable threshold for EBV‐derived next‐generation sequencing reads by mapping them to the EBV genome with three different random arbitrary alignments. We analyzed the prognostic impact of EBV status on the histopathological subtypes of gastric cancer. EBV‐positive cases identified by reanalysis comprised nearly half of the cases (49.6%) independent from infiltrating lymphocyte signatures, and showed significantly longer overall survival for adenocarcinomas of the ‘not‐otherwise‐specified’ type [*P = *0.016 (log‐rank test); hazard ratios (HR): 0.476; 95% CI: 0.260–0.870, *P = *0.016 (Cox univariate analysis)], but shorter overall survival for the tubular adenocarcinoma type [*P = *0.005 (log‐rank test); HR: 3.329; 95% CI: 1.406–7.885, *P = *0.006 (Cox univariate analysis)]. These results demonstrate that the EBV positivity rates were higher when determined by RNA‐seq than when determined by EBER1/2 *in situ* hybridization. The RNA‐seq‐based EBV positivity demonstrated distinct results for gastric cancer prognosis depending on the histopathological subtype, suggesting its potential to be used in clinical prognoses.

## Graphical abstract

We reanalyzed the large‐scale RNA sequencing (RNA‐seq) dataset of gastric cancer (The Cancer Genome Atlas‐Stomach Adenocarcinoma). Epstein–Barr virus (EBV)‐positive cases identified by reanalysis encompassed almost half of the cases, and showed longer overall survival for adenocarcinomas of the ‘not‐otherwise‐specified’ type, but shorter overall survival for the intestinal type. Thus, the RNA‐seq‐based EBV positivity might be useful in clinical prognoses.

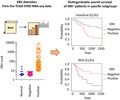

**Abbreviations**
EBV: Epstein–Barr virus
EBVaGC: EBV‐associated GC
GC: gastric cancer
IPW: inverse probability weighting
NGS: next‐generation sequencing
RNA‐seq: RNA sequencing
STAD: Stomach Adenocarcinoma

Epstein–Barr virus (EBV), formally designated as human herpesvirus 4, whose genome length is ~ 172 kbp, infects ~ 90% of *Homo sapiens* worldwide and establishes a lifelong persistent infection, typically with no observable symptoms in immunocompetent hosts 1, 2, 3, 4. Memory B cells are usually the host cell species responsible for establishing latent infections 4. Interestingly, EBV can be detected in lymphoma cells that originate from B cells, and in the rare type of lymphomas originating from T cells or NK cells, nasopharyngeal cancer, and gastric cancer (GC) 3, 4, suggesting that EBV is capable of infecting normal and/or malignant cells, including B, T, NK, and epithelial cells. Numerous studies on EBV have shown carcinogenic activities in EBV genome‐encoded products 4, 5. Hypermethylation of tumor suppressor genes has been observed in EBV‐associated cancers 4, 6, 7, 8, suggesting that aberrant methylation might be a critical mechanism of EBV‐related tumorigenesis.

Gastric cancer is a heterogenous disease, with subtypes, such as diffuse or intestinal, generally being based on histopathological criteria 9. EBV is detected in tumor cells in 2–20% of GC cases 5, 7, and most cases of classical EBV‐associated (EBVa) GC exhibit diffuse histology accompanied by lymphocyte accumulation, which is known as lymphoepithelioma‐like carcinoma 10, 11. However, EBV‐associated GC (EBVaGC) includes other histological subtypes 12, indicating its intrinsic heterogeneity.

Classical EBVaGC has traditionally been diagnosed via *in situ* hybridization, which detects the presence of EBV genome‐derived EBV‐encoded RNA (EBER)1/2 small RNAs in GC cells 13, 14. Based on molecular evidence, as outlined by The Cancer Genome Atlas (TCGA) Stomach Adenocarcinoma (STAD) study 15, four molecular subtypes of GC have been proposed, including tumors positive for EBV, microsatellite‐unstable tumors, genomically stable tumors, and tumors with chromosomal instability. The EBV‐positive subtype in the TCGA‐STAD study was determined by pairwise comparisons of EBV read counts obtained using four sequencing platforms [whole genome, exome, RNA sequencing (RNA‐seq), and microRNA‐seq] but not by EBER *in situ* hybridization 15, 16. Despite the discordant results for EBV positivity among these sequencing methods, positivity was defined by dichotomous values from pairwise plots between two sequencing methods without careful consideration of the small number of sequencing reads 15. Although the concordance between EBV positivity obtained by the TCGA methods and *in situ* hybridization has been validated 16, it remains unclear whether the small amounts of EBV sequencing reads are sufficient for determining prognosis. Additionally, in some reanalysis studies of the TCGA RNA‐seq data, the cutoff levels of next‐generation sequencing (NGS) reads for EBV positivity were determined by arbitrary criteria that excluded small amounts of EBV reads 17, 18, 19.

Several studies have shown that the overall survival (OS) for EBVaGC is longer than in other types of GC that are not associated with EBV 12, 20, 21. However, it has shown that the prognosis of EBVaGC is identical 22, 23, 24, 25, or shorter 26, 27. The first report based on the TCGA‐STAD dataset showed that GC prognosis did not differ between the EBV molecular subtype and the other three subtypes 15. However, when a gene expression data‐based subtype prediction model was used to define the EBV molecular subtype, this subtype was associated with a better prognosis in two large independent cohorts 28. The underlying cause of these discrepancies has yet to be determined.

Recently, Liu *et al*. 29 reported a high‐quality validated survival outcome based on the entire TCGA dataset: The median follow‐up time of STAD was calculated as 14.0 months. Like other TCGA datasets, the OS data from the STAD study were used for survival analysis. Furthermore, ‘primary diagnosis’ data corresponding to histopathological information in the clinical TCGA datasets were harmonized with the terms in the WHO International Classification of Diseases for Oncology, 3rd Edition (ICD‐O‐3; GDC data release 13.0 on September 27, 2018), which allowed us to perform analysis based on current histopathological classifications, rather than on the Lauren scheme. We thus reanalyzed the TCGA‐STAD RNA‐seq data using our own analysis pipeline and successfully obtained wide‐ranging EBV‐derived NGS reads. The reliability of a small number of these reads was examined with the randomness properties of the mapping tool. Considering the heterogeneity of EBVaGC, we performed survival analysis on the EBV‐positive and EBV‐negative groups based on the high‐quality survival outcome 29 and the updated histopathological diagnosis data.

## Materials and methods

### Ethics statement

This study was approved by the Ethics Review Board of Tokyo Metropolitan Institute of Medical Science (IRB#18‐18) and the Tokyo Metropolitan Cancer and Infectious Diseases Center Komagome Hospital (IRB#1563). Approval for access to anonymized RNA‐seq BAM files was obtained from dbGaP 30.

### TCGA‐STAD data and molecular subtype

Sequence data in BAM file format (407 files) and accompanying clinical data were downloaded from the GDC data portal (https://portal.gdc.cancer.gov). We then linked the obtained files using patient identifiers. Among these files, 375 files were derived from tumor samples and the rest from normal samples. BAM files were converted into FASTQ with bam2fastq (https://github.com/jts/bam2fastq). The data used were from cancer specimens and noncancer control specimens.

### EBV detection

We used hg38, constructed by the Genome Reference Consortium, as a human genome reference sequence. We utilized virus detection methods for cancer RNA‐seq data as described elsewhere 17, 18, 31, 32. Briefly, reads that were not mapped to the human genome reference sequence were excised from the BAM files and mapped to virus references. Raw sequencing reads were mapped to reference human genome hg38 with star v.2.4.2a 33, and unmapped reads from the first step were again mapped to hg38 with bowtie2 v.2.2.8 34 using the ‘‐‐very‐sensitive‐local’ option to perform high sensitivity mapping. After trimming low‐quality bases and adaptor sequences with cutadapt v.1.8.1 35, unmapped reads from the previous step were mapped to the virus reference FASTA files listed in Table S2 using bowtie2 with the ‘‐‐very‐sensitive‐local’ option. samtools v.1.8 36 was used to convert the SAM file to BAM and sort the resulting BAM file.

### Verification of EBV reads

In order to verify the small number of detected EBV reads obtained from the analysis pipeline in this study, we implemented our pipeline three times for all samples using the ‘‐‐non‐deterministic’ option of bowtie2, which yielded different results due to the arbitrary choice of alignment (http://bowtie-bio.sourceforge.net/bowtie2/manual.shtml).

### Visualization of EBV reads

Mapped EBV reads were visualized using the Integrative Genomics Viewer 37 with the EBV reference genome.

### Evaluation of lymphocyte infiltration into tumors

We obtained TCGA‐Pan‐Cancer Clinical Data, including extended prognostic data from the supplementary information of a previous report 29 and TCGA immunogenomics data reported in the supplemental table in Thorsson *et al*. 38, which includes intratumoral infiltrating lymphocyte data and intratumoral immune cell scores estimated using cibersort 39.

### Statistical analysis

Statistical analyses were performed with r (The R Foundation for Statistical Computing, Vienna, Austria). In multigroup comparisons, we used the Kruskal–Wallis test with a *post hoc* Dunn's test to estimate the difference between two groups. To test for correlation between EBV read and intratumoral immune cell scores, we used the Spearman correlation coefficients. Survival curves with mortality hazard ratios (HRs) were generated using the Kaplan–Meier method and the univariate Cox proportional hazard model unless otherwise mentioned, applying inverse probability weighting (IPW) in order to adjust for potential imbalances due to confounding factors 40, 41. Propensity scores for calculating IPW were obtained from logistic regression using a set of covariates deemed likely to have affected the outcome, including age, gender, race, tissue or organ of origin, and tumor stage. We acknowledge that there may have been other differences that the TCGA‐STAD project did not measure. The log‐rank test was used to estimate statistical significance in survival analyses.

## Results

### Reanalysis of TCGA‐STAD RNA‐seq data and threshold of EBV reads

**Figure 1:**
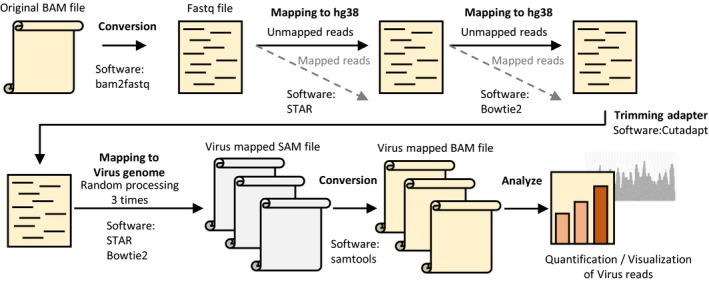
Scheme for detection of viral sequences in TCGA‐STAD RNA‐seq data. Analysis pipeline for detecting viral sequence reads. Each mapped read of EBV was normalized by the total reads mapped to the human genome.

**Figure 2:**
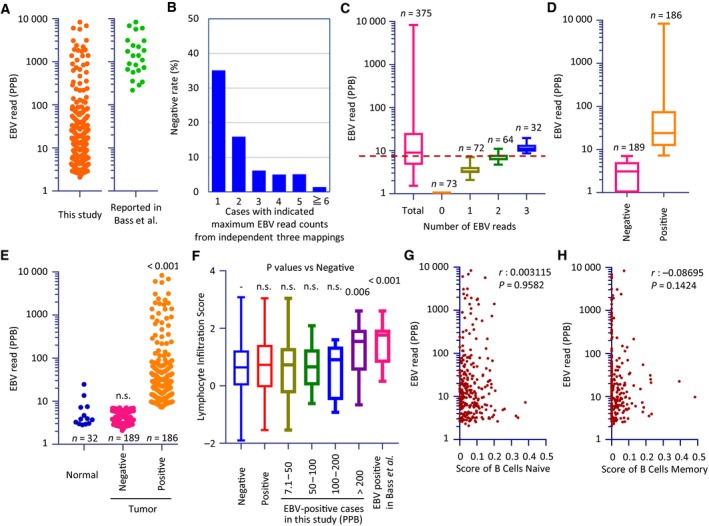
Optimizing the EBV threshold value and scores of tumor‐infiltrating lymphocytes. (A) EBV reads detected in this study (left) and the original report (right). (B) The ambiguity of EBV read counts. Three independent bowtie2 mappings to the EBV genome were implemented for all samples with an option (‐‐non‐deterministic) that led to different results due to the arbitrary choice of alignments (http://bowtie-bio.sourceforge.net/bowtie2/manual.shtml). *X*‐axis values show the detected maximum values of EBV reads. (C) Normalized EBV expression levels in all cases and in cases where EBV expression level ranges from 0 to 3. The dotted line indicates a threshold corresponding to the maximum value of cases in which only one EBV read was detected. (D) Dot plots of normalized EBV reads in normal and tumor samples. A newly defined threshold was applied. (E) Comparison of EBV reads in normal and tumor samples. (F) Lymphocyte infiltration scores in cases grouped by EBV status and each corrected EBV read range. To estimate the statistical difference, the Kruskal–Wallis test with a *post hoc* Dunn's test was used. Scatter plots of EBV reads, and scores of intratumoral naïve B cells (G) or intratumoral memory B cells (H) are shown. Box plots show the median (central line), first and third quartiles (box), and minimum and maximum values (whiskers above and below the boxes, respectively). The Spearman correlation coefficients were calculated for testing correlation between EBV read and intratumoral immune cell scores.

Clinicopathological characteristics of patients in the TCGA‐STAD dataset are shown in Table S1. We first mapped TCGA‐STAD RNA‐seq reads to the human genome and obtained nonhuman reads; the analysis pipeline is shown in Fig. 1. Unmapped reads were mapped to the EBV reference genomes listed in Table S2. Figure 2A shows the number of EBV‐positive cases both in the TCGA‐STAD study and in our analysis. Our analysis pipeline detected EBV in 49.6% cases, in contrast to the TCGA‐STAD frequency of 9% (Fig. 2A). Cumulative transcriptional profiling of the EBV genome revealed EBV reads that were in accordance with a previous report (Fig. S1) 15. To confirm the reliability of the small number of EBV reads in this study, we focused on the mapping process of our analysis pipeline by using bowtie2, which employs a random process (bowtie2 manual, URL: http://bowtie-bio.sourceforge.net/bowtie2/manual.shtml) that yields different results that are filtered out for each calculation. To exclude ambiguous mapping results, we carried out three rounds of mapping with bowtie2 for all samples; Fig. 2B shows the proportion of results in which EBV was absent. The proportion of cases with negative results was the highest (35%) when the maximum EBV read count was 1 (Fig. 2B). On the other hand, if the maximum count of EBV reads was 2 or more, the proportion of negative results was 16% and 5%, respectively. Figure 2C shows corrected EBV read counts with respect to the total read counts in each case (particles per billion reads, ppb). The maximum corrected EBV level of one‐read cases was equivalent to approximately half that of the two‐read cases and was lower than that of all three‐read cases. We thus provisionally adopted the maximum level in one‐read cases as the threshold (7.1 ppb) (Fig. 2C), which resulted in the identification of 186 EBV‐positive cases and 189 EBV‐negative cases (Fig. 2D). Furthermore, we analyzed the normal sample BAM files in TCGA‐STAD using the same analysis pipeline (Fig. 2E). The obtained EBV reads from the normal samples were comparable to those from the EBV‐negative tumor samples. The number of EBV reads from EBV‐positive tumor samples was significantly higher than that of the normal samples and EBV‐negative tumor samples (*P < *0.001). The lymphocyte infiltration score of EBV‐positive cases in the TCGA‐STAD report was significantly higher than that of negative cases. The lymphocyte infiltration scores in the present study, excluding positive cases from the TCGA‐STAD report and > 200 ppb cases in this study, did not differ from those of the negative cases (Fig. 2F). Available intratumoral immune cell scores showed that there was no correlation between EBV reads and the number of intratumoral B‐cell indicators 38 (Fig. 2G,H). We further analyzed the correlation between EBV reads and CD4^+^ T‐cell (Fig. S2A), CD8^+^ T‐cell (Fig. S2B), and natural killer (NK) cell (Fig. S2C) scores, but no correlation was found.

### Survival analysis in reclassified groups according to histopathological type

**Figure 3:**
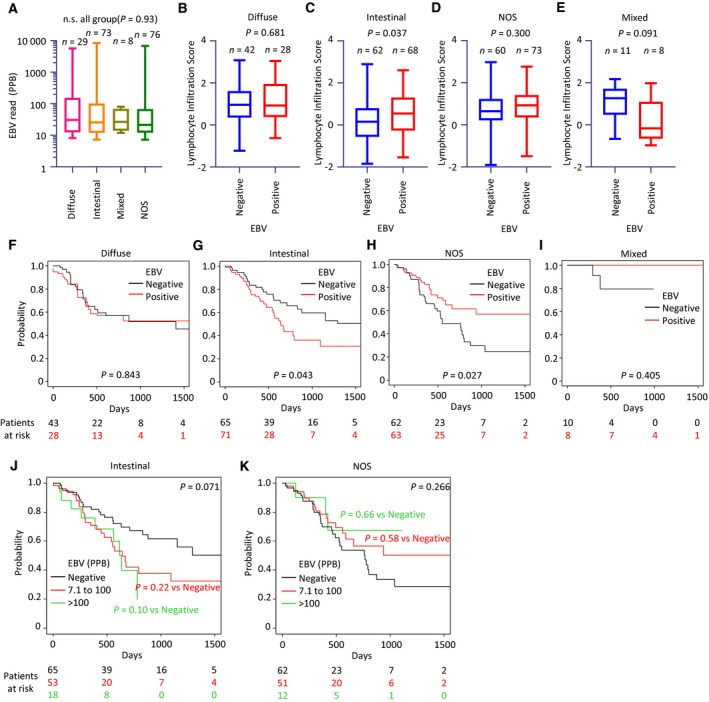
Characteristics of EBV‐positive and EBV‐negative cases classified according to provisional histopathological categories. (A–E) Normalized EBV expression levels in histopathological categories. Lymphocyte infiltration scores in patients with EBV‐negative or EBV‐positive GC, including diffuse (B), intestinal (C), NOS (D), or mixed (E) types. Box plots show the median (central line), first and third quartiles (box), and minimum and maximum values (whiskers above and below the boxes). (F–I) The Kaplan–Meier plots of patients distinguished by RNA‐seq‐based EBV positivity in cases showing four differentiated types. Covariates were adjusted by IPW. (J, K) The Kaplan–Meier plots of patients classified according to the number of EBV reads.

We performed a Kaplan–Meier survival analysis of whole cases. Covariates were adjusted by IPW. There was no difference in adjusted OS between the EBV‐positive and EBV‐negative groups in this study [*P = *0.956 (log‐rank test); HR: 1.008, 95% confidence interval (CI) 0.723–1.406, *P = *0.961 (Cox univariate analysis); Fig. S3A]. We confirmed that there were no differences between the molecular EBV subtype and others, as previously reported 15 (Fig. S3B). Since EBVaGC is associated with diffuse‐type GC 42, 43, 44, we examined whether EBV positivity in each histopathological type affects survival. According to the criterion of whether a luminal structure (i.e., intestinal) was involved, we provisionally reclassified the ICD‐O‐3‐harmonized classification into diffuse, intestinal, mixed, and not‐otherwise‐specified (NOS) types (Table S3). This reclassification is used hereinafter. The average number of EBV reads in EBV‐positive cases did not differ among the reclassified pathological groups (Fig. 3A). Lymphocyte infiltration scores 38 were higher in the diffuse type of EBV‐positive GC than in the intestinal type, irrespective of age (Fig. S4). Statistically significant differences in lymphocyte infiltration scores were observed between EBV‐positive and EBV‐negative cases only in the intestinal type (Fig. 3B–E).

In the Kaplan–Meier survival analysis, EBV positivity demonstrated an unfavorable prognostic effect in the intestinal type [*P = *0.043 (log‐rank test); HR 1.736; 95% CI: 1.019–2.958, *P = *0.043 (Cox univariate analysis); Fig. 3G] but a favorable prognostic effect in the NOS type [*P = *0.027 (log‐rank test); HR 0.520; 5% CI: 0.2907–0.9291, *P = *0.027 (Cox univariate analysis); Fig. 3H]. The statistical power in the mixed type was insufficient due to the small sample size (Fig. 3I). The unadjusted Kaplan–Meier curves were comparable to the IPW‐adjusted results described above (Fig. S5). To validate the prognostic effects of a small number of EBV reads, we carried out a Kaplan–Meier survival analysis for groups classified by the number of EBV reads: those with a small number (7.1–100 ppb) and a large number (more than 100 ppb) of reads. Unlike in Fig. 3G,H, there was no statistically significant difference between the two groups, likely due to the reduction in sample size (Fig. 3J,K). In multivariate Cox survival analysis, the EBV‐positive group with a small number of reads (7.1–100 ppb) showed favorable prognosis in NOS‐type GC, as shown in Fig. 3H (HR: 0.528, 95% CI: 0.283–0.983, *P = *0.044) (Table 1).

**Table 1:** Multivariate Cox survival analysis in patient groups distinguished by the amount of EBV reads.

EBV reads	HR	95% CI	*P*
Intestinal			
Negative	1.000		
7.1–100	1.634	0.923–2.900	0.092
> 100	2.103	0.956–4.627	0.065
NOS			
Negative	1.000		
7.1–100	0.528	0.283–0.983	0.044*
> 100	0.489	0.161–1.485	0.207

* *P* < 0.05.

### Another histopathological criterion: an analysis by TCGA‐provided tumor grade in combination with the provisional histopathological groups

Another histopathology‐based criterion, ‘tumor grade’, is provided by TCGA‐STAD. This information is in the TCGA‐STAD clinical data and shows the degree of abnormality of cancer cells, including a numeric histological grade (G1, well differentiated; G2, moderately differentiated; G3, poorly differentiated; and GX, grade cannot be assessed). Thus, we employed this grade as an alternative classifier in addition to our provisional histopathological categories. Figure S6 shows distinct proportions of G1–GX in the provisional histopathological categories with their original ICD‐O‐3‐harmonized histopathological types, suggesting a difference between the histopathological classification and tumor grade.

**Figure 4:**
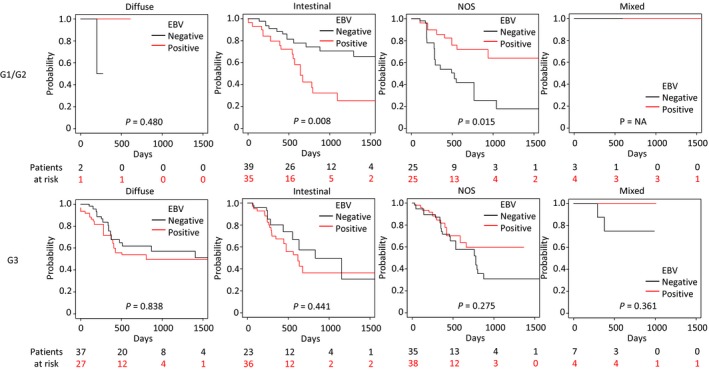
Prognostic effects of EBV‐positive and EBV‐negative cases classified according to tumor grade and the provisional histopathological categories. The Kaplan–Meier plots of patients distinguished by EBV positivity in cases showing the provisional histopathological categories (diffuse, intestinal, NOS, and mixed) and tumor grades (G1/G2, upper; and G3, lower). Covariates were adjusted by IPW. Differences between survival curves were tested for significance by the log‐rank test.

The Kaplan–Meier curves in Fig. 3F–I were further subdivided by G1/G2 (well/moderately differentiated) and G3 (poorly differentiated). Statistically significant differences in prognosis (shorter OS in intestinal, longer OS in NOS) were observed only in G1/G2 (*P = *0.008, 0.015, respectively) but not in G3 (*P = *0.441, 0.275, respectively; Fig. 4). The unadjusted Kaplan–Meier curves (Fig. S7) were comparable to the IPW‐adjusted results (Fig. 4).

We continued to investigate the correlation between EBV reads and tumor grade. There was no statistically significant difference between G1/G2 and G3 based on the number of EBV reads (Fig. S8A). The lymphocyte infiltration score also demonstrated no significant difference between EBV‐negative and EBV‐positive samples in G1/G2 and G3 (Fig. S8B,C). The Kaplan–Meier survival analysis demonstrated no difference in OS between the EBV‐positive and EBV‐negative groups in this study both in G1/G2 and in G3, regardless of IPW adjustment (Fig. S8D–I).

### Survival analysis in original ICD‐O‐3‐harmonized histopathological types with TCGA‐provided tumor grade

**Figure 5:**
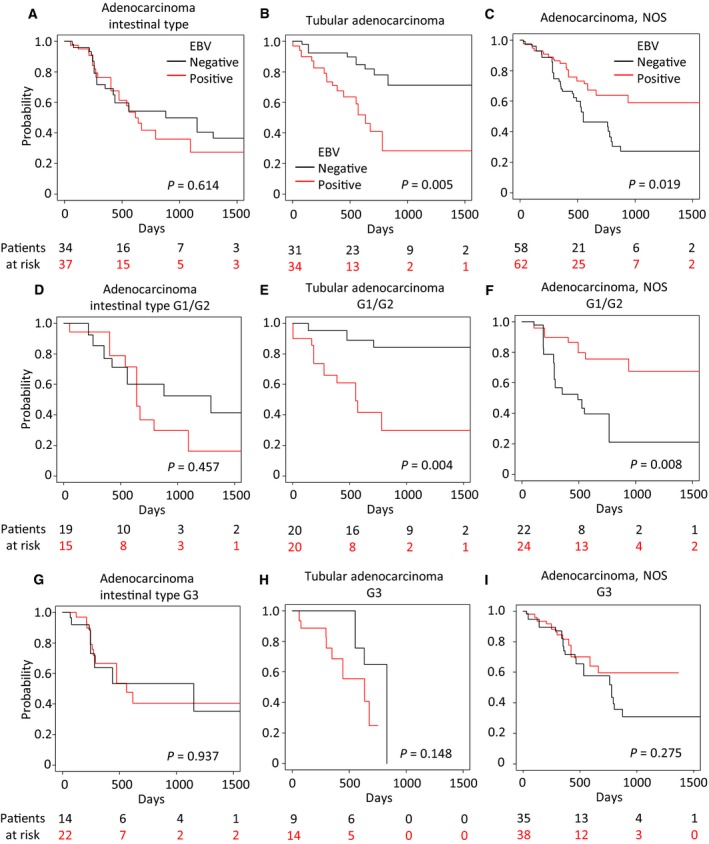
Survival analysis of EBV‐positive and EBV‐negative cases stratified by histopathological features (ICD‐O‐3‐harmonized) and the tumor grade. The Kaplan–Meier plots of patients distinguished by RNA‐seq‐based EBV positivity in cases stratified by ICD‐O‐3‐harmonized histopathology [adenocarcinoma intestinal type (A, D, G), tubular adenocarcinoma (B, E, H), and adenocarcinoma NOS (C, F, I)]. Groups stratified by tumor grade are shown in the middle row (G1/G2) and the lower row (G3). Covariates were adjusted by IPW. *P* values were determined with the log‐rank test.

We next analyzed the prognosis of the original ICD‐O‐3‐harmonized histopathological types that correspond to intestinal and NOS types. Of the assigned ICD‐O‐3‐harmonized histopathological types in each case, we excluded ‘Adenocarcinoma with mixed type’ and ‘Papillary adenocarcinoma NOS’ because of their extremely small sample sizes (*n* = 1 and 5, respectively). Survival analysis was performed for the remaining cases after adjusting for explanatory variables, although some types had low statistical power as a result of their very small sample size (mucinous adenocarcinoma, *n* = 19; signet ring cell carcinoma, *n* = 12). For ‘tubular adenocarcinoma’ (*n* = 65), which corresponds to intestinal‐type GC, EBV‐positive cases demonstrated significantly shorter OS [*P = *0.005 (log‐rank test); HR 3.329; 95% CI: 1.406–7.885, *P = *0.006 (Cox univariate analysis); Fig. 5B], but this was not the case for the ‘adenocarcinoma intestinal’ type (*n* = 71; Fig. 5A). For ‘Adenocarcinoma NOS’ (*n* = 120), EBV‐positive cases showed significantly longer OS [*P = *0.016 (log‐rank test); HR: 0.476; 95% CI: 0.260–0.870, *P = *0.016 (Cox univariate analysis); Fig. 5C]. As seen in the analysis of provisional histopathological categories (Fig. 4), these statistically significant differences in prognoses were observed only in G1/G2 (*P = *0.004, 0.008, respectively) but not in G3 (*P = *0.148, 0.275, respectively; Fig. 5D–I). The unadjusted Kaplan–Meier curves were comparable to the IPW‐adjusted results (Fig. S9A–I). For the ‘carcinoma diffuse’ type (*n* = 59), which was placed in the diffuse classification, we observed no difference between EBV‐positive and EBV‐negative groups [*P = *0.376 (log‐rank test); HR: 0.416, 95% CI: 0.156–1.109, *P = *0.079 (Cox univariate analysis); Fig. S10A]. In the ‘signet ring cell carcinoma’ and the ‘mucinous adenocarcinoma’ types, we were unable to obtain statistically meaningful results due to the small sample size and because patients were censored early on (Fig. S10B,C). The unadjusted Kaplan–Meier curves were once again comparable to the IPW‐adjusted results (Fig. S10A–C).

## Discussion

This is the first report to examine the reliability of EBV reads in very small quantities and the prognostic impact of RNA‐seq‐determined EBV positivity. Our reanalysis of the TCGA‐STAD RNA‐seq dataset yielded a wide range of EBV reads, irrespective of the degree of intratumoral lymphocyte infiltration. EBV positivity had contrasting prognostic effects on the ‘tubular adenocarcinoma’ and ‘adenocarcinoma NOS’ types of GC, which may explain previous conflicting reports on the prognostic value of EBV in GC.

RNA‐seq‐based EBV‐positive GC may exist as a distinct entity that includes EBER1/2 *in situ* hybridization‐positive EBVaGC. The 9% positivity rate of EBV in the original TCGA‐STAD study 15 or the 7–10% as determined by *in situ* hybridization 45, 46 were much lower than the 49.6% observed here and the ~ 40% in another study 47 that were obtained by reanalysis of a portion of the TCGA‐STAD data. These variable results suggest that the assignment of an appropriate cutoff value is essential and that the proportion of EBVaGC is higher than previously recognized. We should note that the definition of EBV positivity depends on the EBV detection method—for example, EBER1/2 *in situ* hybridization vs. RNA‐seq. The latter detects various RNA species derived from the EBV genome, but not small RNAs such as EBER1/2. On the other hand, EBER1/2 *in situ* hybridization detects a predetermined target and may only detect small RNAs when there are a sufficient number of copies. There have been no previous reports that EBER expression is necessary and sufficient for confirming the existence of EBV in cancer tissue.

In our study, we could not detect any correlation between EBV transcripts and intratumoral immune scores of T cells, B cells, and NK cells, which are the primary or potential hosts of latent EBV 4, suggesting that EBV reads were not derived from these intratumoral immune cells. These results also imply that EBV reads could be obtained from tumor cells, even at low read counts. The small number of EBV reads in tumor tissues may be partly explained by the hit‐and‐run hypothesis of herpesvirus 48, 49, 50, which posits that the virus inflicts genetic or epigenetic injury to infected cells. The agreement between the reliable results obtained from repeated stochastic mapping and the transcriptional profile of the EBV genome 15 (Fig. S1) allowed us to consider the EBV reads in this study as actual EBV transcripts. To validate the method for both false‐positive and false‐negative EBV transcripts in the future, an internal standard RNA sample that includes the relevant EBV transcripts may be a prerequisite for EBV detection in cancer tissues.

The high rate (46.9%) of RNA‐seq‐based EBV positivity in this study may be useful for estimating GC prognosis. We have shown that the prognoses of cases with EBV reads were poor in the tubular adenocarcinoma type of GC, but were improved in adenocarcinoma NOS. The variable prognostic significance of EBV positivity may be attributable to the specific genetic and/or epigenetic features that underlie tumor histopathology and the immune response. Given the inconsistency between the G1/G2/G3 scale and the ICD‐O‐3‐harmonized histopathological type for ‘adenocarcinoma NOS’ (Figs 4 and 5, and Fig. S6), this GC type is most likely intrinsically heterogeneous and may be further classified by distinct tumor biology and immune features. Additional studies are needed to substantiate and confirm this at the molecular level.

It is important to note that the GC classifications made by the Lauren and WHO schemes are discordant. According to the Lauren classification, GCs are separated into two main histological types, diffuse and intestinal, in addition to the mixed and indeterminate types. In this study, we recategorized eight ICD‐O‐3‐derived groups into four new groups: diffuse, intestinal, NOS, and mixed (Table S3). These groups resemble but are not identical to the Lauren scheme. As such, extrapolation of the results of this study to GC studies performed in different pathological contexts must be done with caution.

Additionally, while IPW adjustment can reduce the impact of potentially confounding factors, it is subject to biases from unobserved differences. Furthermore, it cannot be ruled out that EBV read detection is due to systematic contamination from laboratory reagents and processes and/or the environment, although, considering the high EBV infection rate in humans, our results can be regarded as plausible. Moreover, EBV has not been detected in reports on contamination 51, 52, 53. Lastly, EBV positivity requires validation in different cohorts.

## Conclusion

A reanalysis of the TCGA‐STAD RNA‐seq dataset revealed a wide range of EBV reads that are independent of the degree of infiltrating lymphocyte signatures. We found that EBV‐positive cases had variable prognoses depending on their histopathological subtype. Thus, RNA‐seq‐based EBV positivity may be a useful tool for estimating the prognosis of specific histopathological types of GC.

## Conflicts of interest

The authors declare no conflict of interest.

## Author contributions

DS and K Oboki designed the study concept. DS, MO, CH, and K Oboki analyzed the data. K Oboki was responsible for funding procurement. DS, MO, CH, TH, S‐IH, and K Oboki contributed to the investigation. DS and K Oboki contributed to the development of methodology. YH, TS, MI, K Ohashi, and K Oboki underwent supervision. DS and K Oboki contributed to the writing of the manuscript.

## Supporting information


**Fig. S1.** Cumulative transcriptional profiling of the EBV genome. (A) Landscape of EBV reads on EBV Akata strain (KC207813.1). (B) Enlarged genomic region with mapped EBV reads for LF3. (C) Enlarged genomic region with mapped EBV reads for A73, BALF4, and BARF0.

**Fig. S2.** Scatter plots of EBV reads and scores of intratumoral T cells and NK cells. (A) CD4^+^ T cells (memory activated, memory resting, and naïve), (B) CD8^+^ T cells, and (C) NK cells (activated or resting). Spearman correlation coefficients were calculated for testing correlation between EBV read and intratumoral immune cell scores.

**Fig. S3.** Kaplan–Meier plot of whole cases. (A) Survival analysis based on RNA‐seq‐based EBV positivity in the entire patients of this study (left, unadjusted; right, IPW‐adjusted). (B) Survival analysis based on molecular EBV status in all TCGA‐STAD patients (left, unadjusted; right, IPW‐adjusted). *P* values were determined using the log‐rank test.

**Fig. S4.** Lymphocyte infiltration scores in TCGA GC patients reclassified based on histopathological features. Patient groups are as follows: all ages, 35–64 years old, and 65–90 years old. To estimate the statistical difference, Kruskal–Wallis test with a *post hoc* Dunn's test was used.

**Fig. S5.** Unadjusted Kaplan–Meier plot for the four provisional histopathological groups. These Kaplan–Meier plots (A–D) correspond to the data shown in Fig. 3F–I.

**Fig. S6.** Proportion of G1–GX in the provisional histopathological categories (diffuse, intestinal, NOS and mixed types) or their original ICD‐O‐3‐harmonized histopathological types.

**Fig. S7.** Unadjusted Kaplan–Meier curves corresponding to the data shown in Fig. 4. Prognostic effects of EBV‐positive and ‐negative cases classified according to tumor grade and the provisional histopathological categories are shown.

**Fig. S8.** Characteristics of EBV‐positive and ‐negative cases classified according to the TCGA‐provided tumor grade. (A) Normalized EBV expression levels in histopathological categories. Lymphocyte infiltration scores in patients with EBV‐negative or ‐positive GC, in G1/G2 (B), or G3 (C). Box plots show the median (central line), first and third quartiles (box), and minimum and maximum values (whiskers above and below the boxes). To estimate the statistical difference, Kruskal‐Wallis test with a *post hoc* Dunn's test was used. (D–G) Kaplan–Meier plots of patients distinguished by RNA‐seq‐based EBV positivity in G1/G2 or G3 cases. (H, I) Kaplan–Meier plots of patients classified according to the number of EBV reads. Covariates were adjusted by IPW.

**Fig. S9.** Unadjusted Kaplan–Meier curves corresponding to the data shown in Fig. 5. Prognostic effects of EBV‐positive and ‐negative case s classified according to tumor grade and the provisional histopathological categories. Intestinal type [adenocarcinoma intestinal type (A, D, G) and tubular adenocarcinoma (B, E, H)]. NOS type [adenocarcinoma NOS (C, F, I)].

**Fig. S10.** Kaplan–Meier curves of the remaining histopathological types (ICD‐O‐3‐harmonized) not listed in Fig. 5. (A) Carcinoma diffuse type, (B) signet ring cell carcinoma, and (C) mucinous adenocarcinoma with IPW‐adjustment (upper) or without IPW‐adjustment (lower).

**Table S1.** Baseline characteristics of patients in the Cancer Genome Atlas‐Stomach Adenocarcinoma (TCGA‐STAD) dataset.

**Table S2.** List of EBV DNA reference sequences for mapping next‐generation sequencing short reads.

**Table S3.** Provisional regrouped histopathological categories.

## References

[1] Kutok JL and Wang F (2006) Spectrum of Epstein‐Barr virus‐associated diseases. Annu Rev Pathol 1, 375–404. 18039120 10.1146/annurev.pathol.1.110304.100209

[2] Hislop AD , Taylor GS , Sauce D and Rickinson AB (2007) Cellular responses to viral infection in humans: lessons from Epstein‐Barr virus. Annu Rev Immunol 25, 587–617. 17378764 10.1146/annurev.immunol.25.022106.141553

[3] Taylor GS , Long HM , Brooks JM , Rickinson AB and Hislop AD (2015) The immunology of Epstein‐Barr virus‐induced disease. Annu Rev Immunol 33, 787–821. 25706097 10.1146/annurev-immunol-032414-112326

[4] Young LS , Yap LF and Murray PG (2016) Epstein‐Barr virus: more than 50 years old and still providing surprises. Nat Rev Cancer 16, 789–802. 27687982 10.1038/nrc.2016.92

[5] Farrell PJ (2019) Epstein – Barr virus and cancer. Annu Rev Pathol Mech Dis 14, 29–53. 10.1146/annurev-pathmechdis-012418-013023 30125149

[6] Tempera I and Lieberman PM (2014) Epigenetic regulation of EBV persistence and oncogenesis. Semin Cancer Biol 26, 22–29. 24468737 10.1016/j.semcancer.2014.01.003 PMC4048758

[7] Shinozaki‐Ushiku A , Kunita A and Fukayama M (2015) Update on Epstein‐Barr virus and gastric cancer (review). Int J Oncol 46, 1421–1434. 25633561 10.3892/ijo.2015.2856

[8] Scott RS (2017) Epstein‐Barr virus: a master epigenetic manipulator. Curr Opin Virol 26, 74–80. 28780440 10.1016/j.coviro.2017.07.017 PMC5742862

[9] Lauren P (1965) The two histological main types of gastric carcinoma: diffuse and so‐called intestinal‐type carcinoma. An attempt at a histo‐clinical classification. Acta Pathol Microbiol Scand 64, 31–49. 14320675 10.1111/apm.1965.64.1.31

[10] Shibata D and Weiss LM (1992) Epstein‐Barr virus‐associated gastric adenocarcinoma. Am J Pathol 140, 769–774. 1314023 PMC1886378

[11] Cho J , Kang M‐S and Kim K‐M (2016) Epstein‐Barr virus‐associated gastric carcinoma and specific features of the accompanying immune response. J Gastric Cancer 16, 1. 27104020 10.5230/jgc.2016.16.1.1 PMC4834615

[12] Song H‐J , Srivastava A , Lee J , Kim YS , Kim K‐M , Ki Kang W , Kim M , Kim S , Park CK and Kim S (2010) Host inflammatory response predicts survival of patients with Epstein‐Barr virus‐associated gastric carcinoma. Gastroenterology 139, 84–92.e2. 20398662 10.1053/j.gastro.2010.04.002

[13] Tokunaga M , Land CE , Uemura Y , Tokudome T , Tanaka S and Sato E (1993) Epstein‐Barr virus in gastric carcinoma. Am J Pathol 143, 1250–1254. 8238241 PMC1887176

[14] Ambinder RF and Mann RB (1994) Epstein‐Barr‐encoded RNA *in situ* hybridization: diagnostic applications. Hum Pathol 25, 602–605. 8013952 10.1016/0046-8177(94)90227-5

[15] Bass AJ , Thorsson V , Shmulevich I , Reynolds SM , Miller M , Bernard B , Hinoue T , Laird PW , Curtis C , Shen H *et al* (2014) Comprehensive molecular characterization of gastric adenocarcinoma. Nature 513, 202–209. 25079317 10.1038/nature13480 PMC4170219

[16] Camargo MC , Bowlby R , Chu A , Pedamallu CS , Thorsson V , Elmore S , Mungall AJ , Bass AJ , Gulley ML and Rabkin CS (2016) Validation and calibration of next‐generation sequencing to identify Epstein‐Barr virus‐positive gastric cancer in The Cancer Genome Atlas. Gastric Cancer 19, 676–681. 26095338 10.1007/s10120-015-0508-x PMC4689675

[17] Tang K‐W , Alaei‐Mahabadi B , Samuelsson T , Lindh M and Larsson E (2013) The landscape of viral expression and host gene fusion and adaptation in human cancer. Nat Commun 4, 2513. 24085110 10.1038/ncomms3513 PMC3806554

[18] Khoury JD , Tannir NM , Williams MD , Chen Y , Yao H , Zhang J , Thompson EJ , Network TCGA , Meric‐Bernstam F , Medeiros LJ *et al* (2013) Landscape of DNA virus associations across human malignant cancers: analysis of 3,775 cases using RNA‐Seq. J Virol 87, 8916–8926. 23740984 10.1128/JVI.00340-13 PMC3754044

[19] Cao S , Wendl MC , Wyczalkowski MA , Wylie K , Ye K , Jayasinghe R , Xie M , Wu S , Niu B , Grubb R *et al* (2016) Divergent viral presentation among human tumors and adjacent normal tissues. Sci Rep 6, 28294. 27339696 10.1038/srep28294 PMC4919655

[20] van Beek J and zur Hausen A , Klein Kranenbarg E , van de Velde CJH , Middeldorp JM , van den Brule AJC , Meijer CJLM and Bloemena E (2004) EBV‐positive gastric adenocarcinomas: a distinct clinicopathologic entity with a low frequency of lymph node involvement. J Clin Oncol 22, 664–670. 14966089 10.1200/JCO.2004.08.061

[21] Camargo MC , Kim W‐H , Chiaravalli AM , Kim K‐M , Corvalan AH , Matsuo K , Yu J , Sung JJY , Herrera‐Goepfert R , Meneses‐Gonzalez F *et al* (2014) Improved survival of gastric cancer with tumour Epstein‐Barr virus positivity: an international pooled analysis. Gut 63, 236–243. 23580779 10.1136/gutjnl-2013-304531 PMC4384434

[22] Gulley ML , Pulitzer DR , Eagan PA and Schneider BG (1996) Epstein‐Barr virus infection is an early event in gastric carcinogenesis and is independent of bcl‐2 expression and p53 accumulation. Hum Pathol 27, 20–27. 8543306 10.1016/s0046-8177(96)90133-1

[23] Chang MS , Lee HS , Kim CW , Kim YI and Kim WH (2001) Clinicopathologic characteristics of Epstein‐Barr virus‐incorporated gastric cancers in Korea. Pathol Res Pract 197, 395–400. 11432666 10.1078/0344-0338-00052

[24] Kijima Y , Ishigami S , Hokita S , Koriyama C , Akiba S , Eizuru Y and Aikou T (2003) The comparison of the prognosis between Epstein‐Barr virus (EBV)‐positive gastric carcinomas and EBV‐negative ones. Cancer Lett 200, 33–40. 14550950 10.1016/s0304-3835(03)00410-5

[25] Huang SC , Ng KF , Chen KH , Hsu JT , Liu KH , Yeh TS and Chen TC (2014) Prognostic factors in Epstein‐Barr virus‐associated stage I‐III gastric carcinoma: implications for a unique type of carcinogenesis. Oncol Rep 32, 530–538. 24899228 10.3892/or.2014.3234

[26] Koriyama C , Akiba S , Itoh T , Kijima Y , Sueyoshi K , Corvalan A , Herrera‐Goepfer R and Eizuru Y (2002) Prognostic significance of Epstein‐Barr virus involvement in gastric carcinoma in Japan. Int J Mol Med 10, 635–639. 12373307

[27] Wang W , Wang K , Chen Z , Chen L , Guo W , Liao P , Rotroff D , Knepper TC , Liu Z , Zhang W *et al* (2018) Immunoclassification characterized by CD8 and PD‐L1 expression is associated with the clinical outcome of gastric cancer patients. Oncotarget 9, 12164–12173. 29552300 10.18632/oncotarget.24037 PMC5844736

[28] Sohn BH , Hwang J‐E , Jang H‐J , Lee H‐S , Oh SC , Shim J‐J , Lee K‐W , Kim EH , Yim SY , Lee SH *et al* (2017) Clinical significance of four molecular subtypes of gastric cancer identified by The Cancer Genome Atlas project. Clin Cancer Res 23, 4441–4449. 10.1158/1078-0432.CCR-16-2211 PMC5785562 28747339

[29] Liu J , Lichtenberg T , Hoadley KA , Poisson LM , Lazar AJ , Cherniack AD , Kovatich AJ , Benz CC , Levine DA , Lee AV *et al* (2018) An integrated TCGA pan‐cancer clinical data resource to drive high‐quality survival outcome analytics. Cell 173, 400–416.e11. 29625055 10.1016/j.cell.2018.02.052 PMC6066282

[30] Wong KM , Langlais K , Tobias GS , Fletcher‐Hoppe C , Krasnewich D , Leeds HS , Rodriguez LL , Godynskiy G , Schneider VA , Ramos EM *et al* (2017) The dbGaP data browser: a new tool for browsing dbGaP controlled‐access genomic data. Nucleic Acids Res 45, D819–D826. 27899644 10.1093/nar/gkw1139 PMC5210596

[31] Wang Q , Jia P and Zhao Z (2013) VirusFinder: software for efficient and accurate detection of viruses and their integration sites in host genomes through next generation sequencing data. PLoS ONE 8, e64465. 23717618 10.1371/journal.pone.0064465 PMC3663743

[32] Chen Y , Yao H , Thompson EJ , Tannir NM , Weinstein JN and Su X (2013) VirusSeq: software to identify viruses and their integration sites using next‐generation sequencing of human cancer tissue. Bioinformatics 29, 266–267. 23162058 10.1093/bioinformatics/bts665 PMC3546792

[33] Dobin A , Davis CA , Schlesinger F , Drenkow J , Zaleski C , Jha S , Batut P , Chaisson M and Gingeras TR (2013) STAR: ultrafast universal RNA‐seq aligner. Bioinformatics 29, 15–21. 23104886 10.1093/bioinformatics/bts635 PMC3530905

[34] Langmead B and Salzberg SL (2012) Fast gapped‐read alignment with Bowtie 2. Nat Methods 9, 357–359. 22388286 10.1038/nmeth.1923 PMC3322381

[35] Martin M (2011) Cutadapt removes adapter sequences from high‐throughput sequencing reads. EMBnet J 17, 10.

[36] Li H , Handsaker B , Wysoker A , Fennell T , Ruan J , Homer N , Marth G , Abecasis G , Durbin R & 1000 Genome Project Data Processing Subgroup (2009) The sequence alignment/map format and SAMtools. Bioinformatics 25, 2078–2079. 19505943 10.1093/bioinformatics/btp352 PMC2723002

[37] Robinson JT , Thorvaldsdóttir H , Winckler W , Guttman M , Lander ES , Getz G and Mesirov JP (2011) Integrative genomics viewer. Nat Biotechnol 29, 24–26. 21221095 10.1038/nbt.1754 PMC3346182

[38] Thorsson V , Gibbs DL , Brown SD , Wolf D , Bortone DS , Ou Yang T‐H , Porta‐Pardo E , Gao GF , Plaisier CL , Eddy JA *et al.* (2018) The immune landscape of cancer. Immunity 48, 812–830.e14. 29628290 10.1016/j.immuni.2018.03.023 PMC5982584

[39] Newman AM , Liu CL , Green MR , Gentles AJ , Feng W , Xu Y , Hoang CD , Diehn M and Alizadeh AA (2015) Robust enumeration of cell subsets from tissue expression profiles. Nat Methods 12, 453–457. 25822800 10.1038/nmeth.3337 PMC4739640

[40] Little RJ and Rubin DB (2000) Causal effects in clinical and epidemiological studies via potential outcomes: concepts and analytical approaches. Annu Rev Public Health 21, 121–145. 10884949 10.1146/annurev.publhealth.21.1.121

[41] Cole SR and Hernán MA (2004) Adjusted survival curves with inverse probability weights. Comput Methods Programs Biomed 75, 45–49. 15158046 10.1016/j.cmpb.2003.10.004

[42] Corvalan A , Koriyama C , Akiba S , Eizuru Y , Backhouse C , Palma M , Argandoña J and Tokunaga M (2001) Epstein‐Barr virus in gastric carcinoma is associated with location in the cardia and with a diffuse histology: a study in one area of Chile. Int J Cancer 94, 527–530. 11745439 10.1002/ijc.1510

[43] Herrera‐Goepfert R , Akiba S , Koriyama C , Ding S , Reyes E , Itoh T , Minakami Y and Eizuru Y (2005) Epstein‐Barr virus‐associated gastric carcinoma: evidence of age‐dependence among a Mexican population. World J Gastroenterol 11, 6096–6103. 16273633 10.3748/wjg.v11.i39.6096 PMC4436624

[44] Carrasco‐Avino G , Riquelme I , Padilla O , Villaseca M , Aguayo FR and Corvalan AH (2017) The conundrum of the Epstein‐Barr virus‐associated gastric carcinoma in the Americas. Oncotarget 8, 75687–75698. 29088902 10.18632/oncotarget.18497 PMC5650457

[45] Camargo MC , Murphy G , Koriyama C , Pfeiffer RM , Kim WH , Herrera‐Goepfert R , Corvalan AH , Carrascal E , Abdirad A , Anwar M *et al* (2011) Determinants of Epstein‐Barr virus‐positive gastric cancer: an international pooled analysis. Br J Cancer 105, 38–43. 21654677 10.1038/bjc.2011.215 PMC3137422

[46] Fukayama M , Kunita A and Kaneda A (2018) Gastritis‐infection‐cancer sequence of Epstein‐Barr virus‐associated Gastric Cancer. Adv Exp Med Biol 1045, 437–457. 29896679 10.1007/978-981-10-7230-7_20

[47] Song H , Lim Y , Im H , Bae JM , Kang GH , Ahn J , Baek D , Kim T‐Y , Yoon S‐S and Koh Y (2019) Interpretation of EBV infection in pan‐cancer genome considering viral life cycle: LiEB (Life cycle of Epstein‐Barr virus). Sci Rep 9, 3465. 30837539 10.1038/s41598-019-39706-0 PMC6401378

[48] Galloway DA and McDougall JK (1983) The oncogenic potential of herpes simplex viruses: evidence for a “hit‐and‐run” mechanism. Nature 302, 21–24. 6298634 10.1038/302021a0

[49] Ambinder RF (2000) Gammaherpesviruses and “Hit‐and‐Run” oncogenesis. Am J Pathol 156, 1–3. 10623645 10.1016/S0002-9440(10)64697-4 PMC1868625

[50] Niller HH , Wolf H and Minarovits J (2011) Viral hit and run‐oncogenesis: genetic and epigenetic scenarios. Cancer Lett 305, 200–217. 20813452 10.1016/j.canlet.2010.08.007

[51] Naccache SN , Greninger AL , Lee D , Coffey LL , Phan T , Rein‐Weston A , Aronsohn A , Hackett J , Delwart EL and Chiu CY (2013) The perils of pathogen discovery: origin of a novel parvovirus‐like hybrid genome traced to nucleic acid extraction spin columns. J Virol 87, 11966–11977. 24027301 10.1128/JVI.02323-13 PMC3807889

[52] Smuts H , Kew M , Khan A and Korsman S (2014) Novel hybrid parvovirus‐like virus, NIH‐CQV/PHV, contaminants in silica column‐based nucleic acid extraction kits. J Virol 88, 1398. 24335290 10.1128/JVI.03206-13 PMC3911631

[53] Friis‐Nielsen J , Kjartansdóttir KR , Mollerup S , Asplund M , Mourier T , Jensen RH , Hansen TA , Rey‐Iglesia A , Richter SR , Nielsen IB *et al* (2016) Identification of known and novel recurrent viral sequences in data from multiple patients and multiple cancers. Viruses 8, 53. 10.3390/v8020053 PMC4776208 26907326

