# Supplementary figures and images for: Potential prognostic impact of EBV RNA‐seq reads in gastric cancer: a reanalysis of The Cancer Genome Atlas cohort
Source: FEBS Open Bio. 2020 Feb 16;10(3):455–67. doi: 10.1002/2211-5463.12803 (PMC7050242; doi:10.1002/2211-5463.12803)

Figure S1

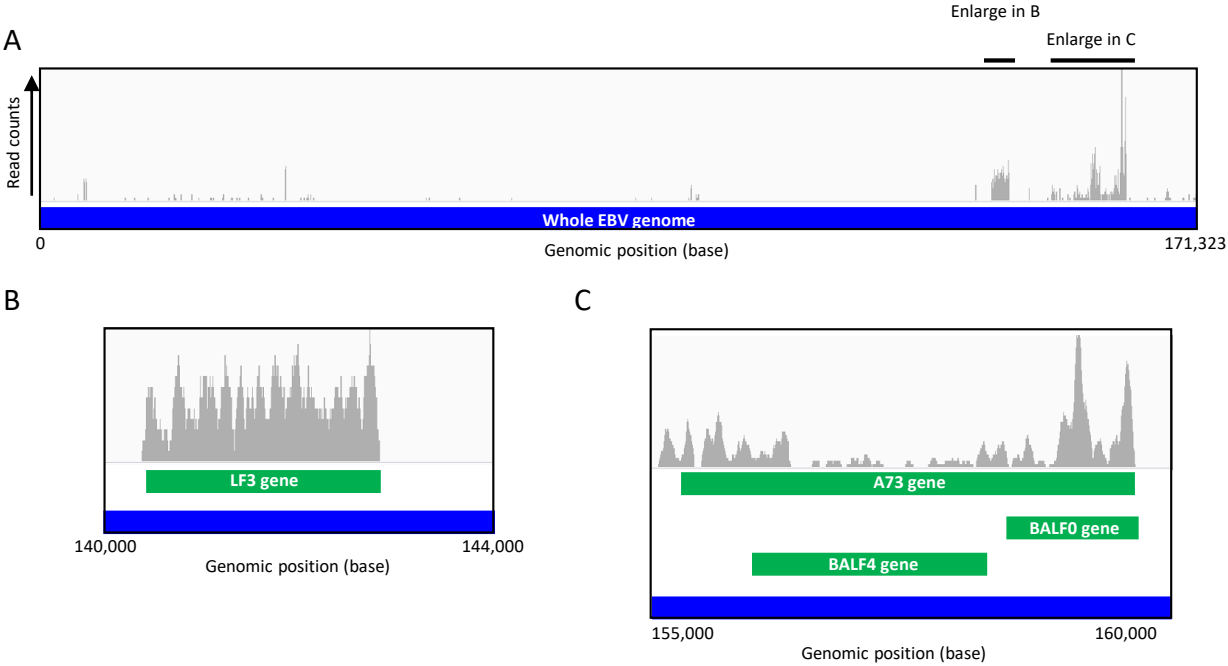

Supplement: Supplementary file 1 — Fig. S1. Cumulative transcriptional profiling of the EBV genome. (A) Landscape of EBV reads on EBV Akata strain (KC207813.1). (B) Enlarged genomic region with mapped EBV reads for LF3. (C) Enlarged genomic region with mapped EBV reads for A73, BALF4, and BARF0. [file FEB4-10-455-s001.pdf]

Figure S2

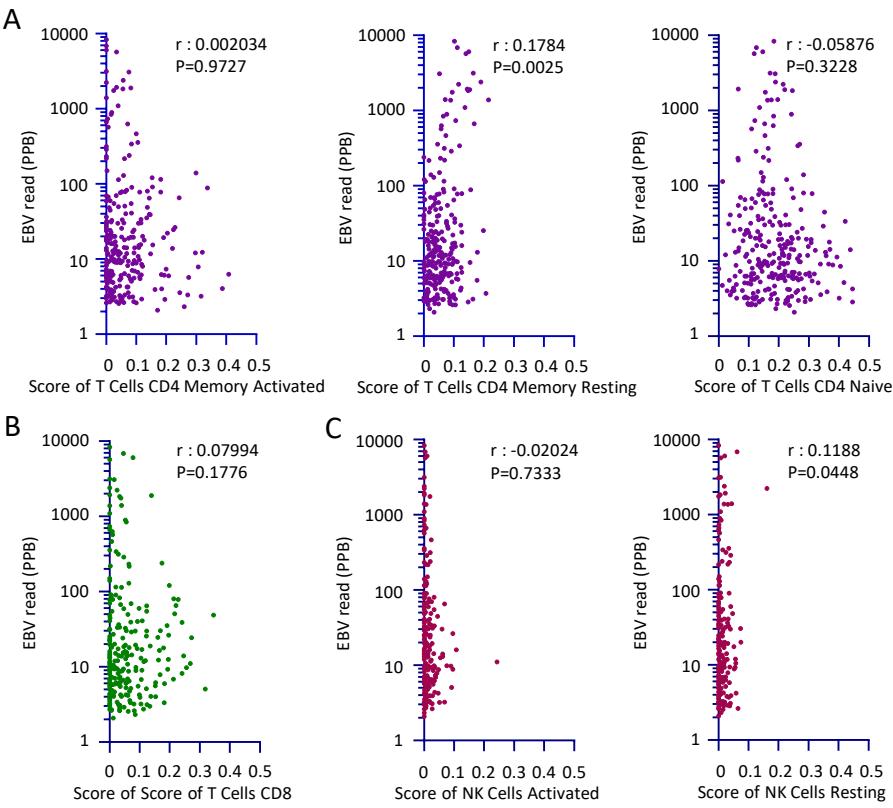

Supplement: Supplementary file 2 — Fig. S2. Scatter plots of EBV reads and scores of intratumoral T cells and NK cells. (A) CD4+ T cells (memory activated, memory resting, and naïve), (B) CD8+ T cells, and (C) NK cells (activated or resting). Spearman correlation coefficients were calculated for testing correlation between EBV read and intratumoral immune cell scores. [file FEB4-10-455-s002.pdf]

Figure S3

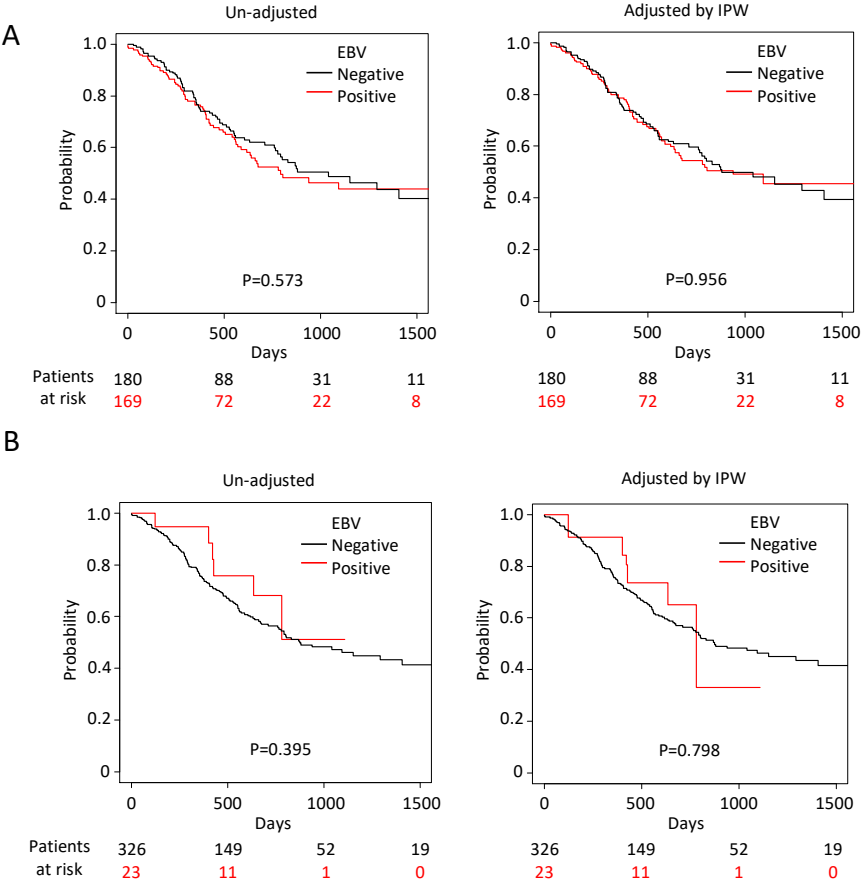

Supplement: Supplementary file 3 — Fig. S3. Kaplan–Meier plot of whole cases. (A) Survival analysis based on RNA‐seq‐based EBV positivity in the entire patients of this study (left, unadjusted; right, IPW‐adjusted). (B) Survival analysis based on molecular EBV status in all TCGA‐STAD patients (left, unadjusted; right, IPW‐adjusted). P values were determined using the log‐rank test. [file FEB4-10-455-s003.pdf]

Figure S4

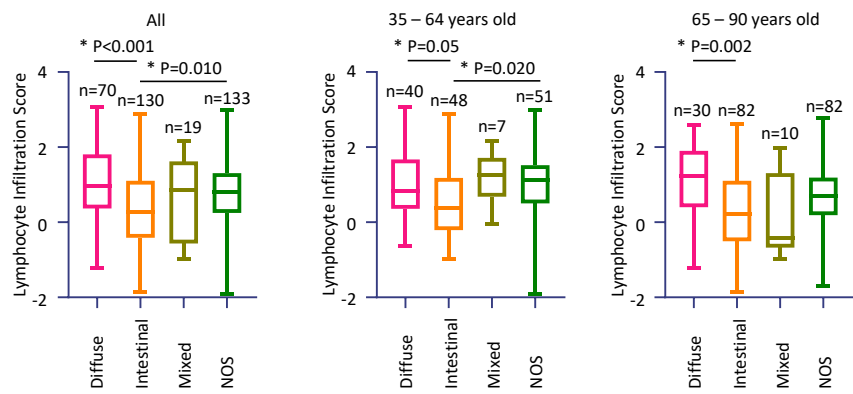

Supplement: Supplementary file 4 — Fig. S4. Lymphocyte infiltration scores in TCGA GC patients reclassified based on histopathological features. Patient groups are as follows: all ages, 35–64 years old, and 65–90 years old. To estimate the statistical difference, Kruskal–Wallis test with a post hoc Dunn's test was used. [file FEB4-10-455-s004.pdf]

Figure S5

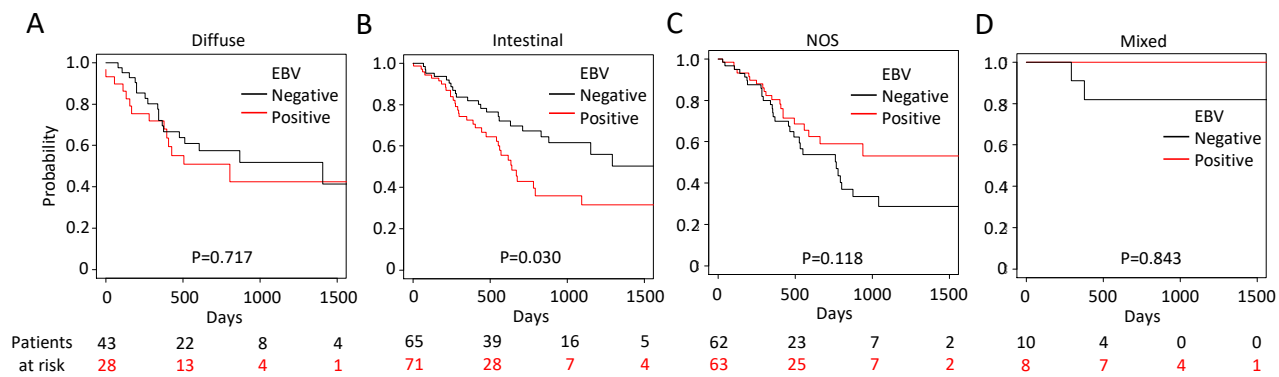

Supplement: Supplementary file 5 — Fig. S5. Unadjusted Kaplan–Meier plot for the four provisional histopathological groups. These Kaplan–Meier plots (A–D) correspond to the data shown in Fig. 3F–I. [file FEB4-10-455-s005.pdf]

Figure S6

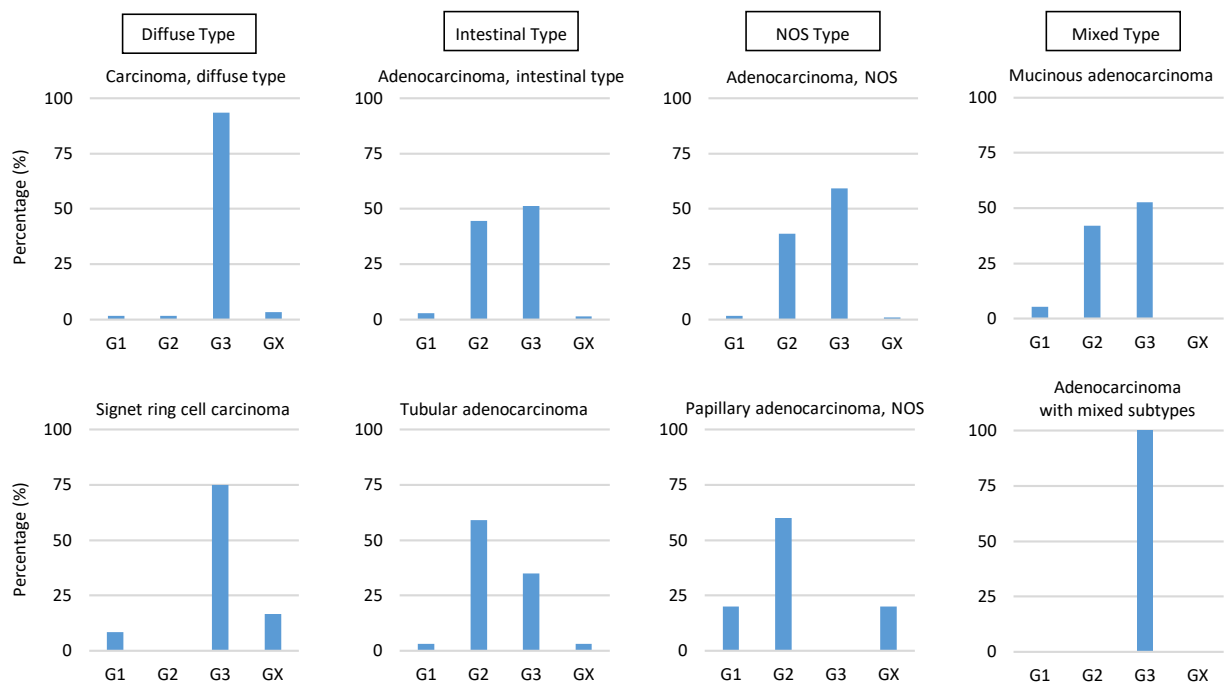

Supplement: Supplementary file 6 — Fig. S6. Proportion of G1–GX in the provisional histopathological categories (diffuse, intestinal, NOS and mixed types) or their original ICD‐O‐3‐harmonized histopathological types. [file FEB4-10-455-s006.pdf]

Figure S7

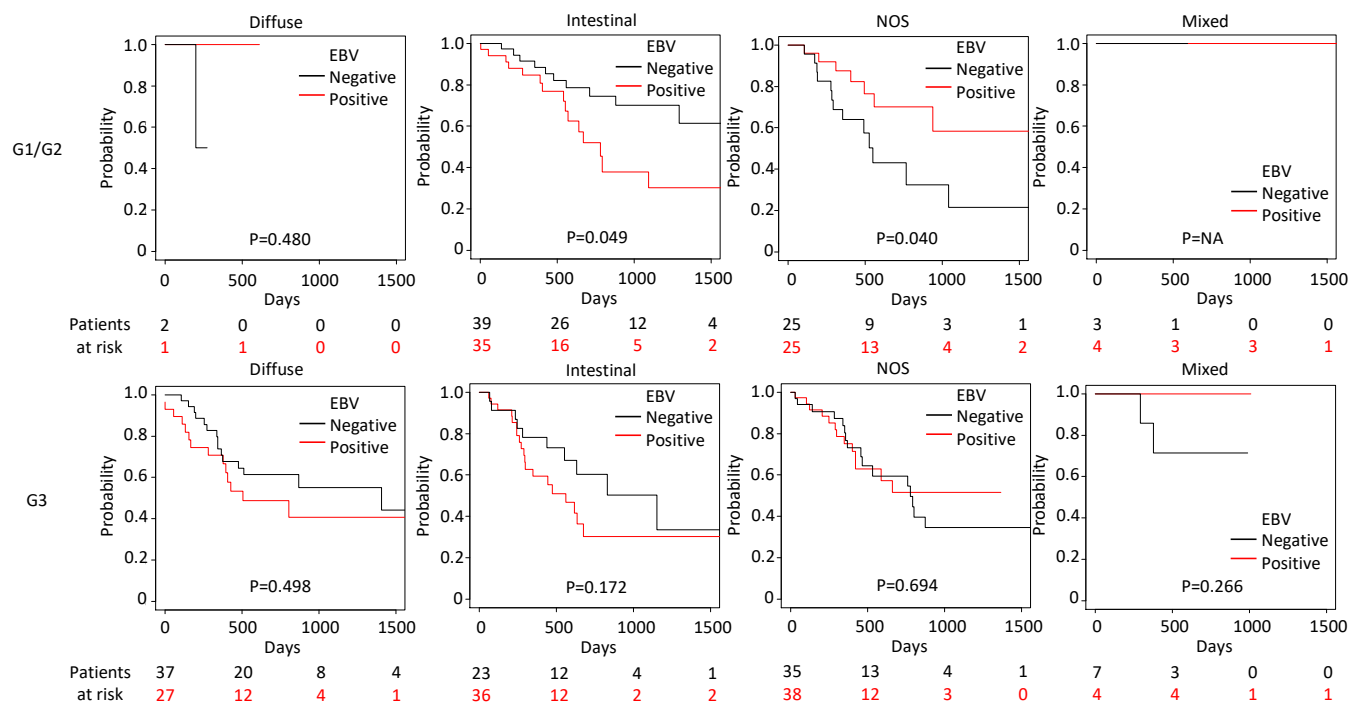

Supplement: Supplementary file 7 — Fig. S7. Unadjusted Kaplan–Meier curves corresponding to the data shown in Fig. 4. Prognostic effects of EBV‐positive and ‐negative cases classified according to tumor grade and the provisional histopathological categories are shown. [file FEB4-10-455-s007.pdf]

Figure S8

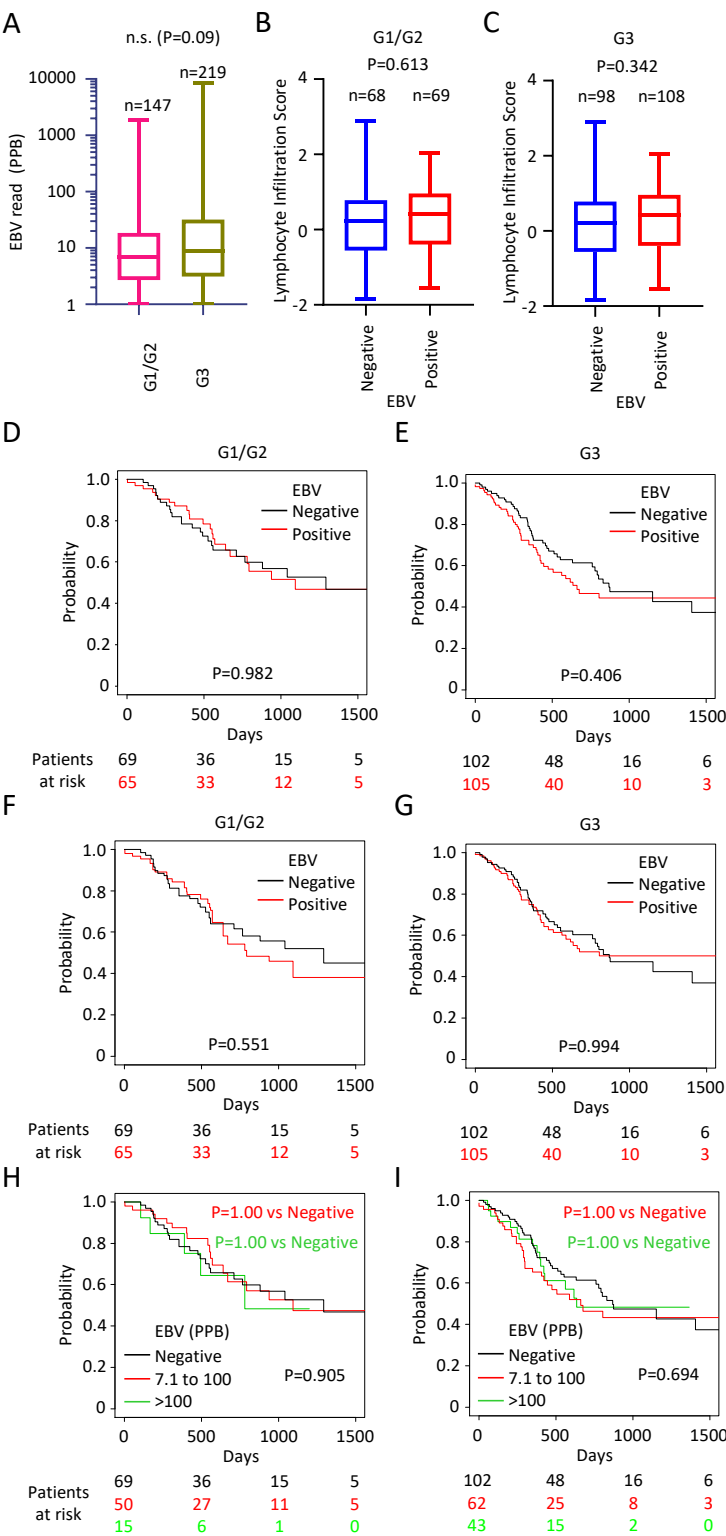

Supplement: Supplementary file 8 — Fig. S8. Characteristics of EBV‐positive and ‐negative cases classified according to the TCGA‐provided tumor grade. (A) Normalized EBV expression levels in histopathological categories. Lymphocyte infiltration scores in patients with EBV‐negative or ‐positive GC, in G1/G2 (B), or G3 (C). Box plots show the median (central line), first and third quartiles (box), and minimum and maximum values (whiskers above and below the boxes). To estimate the statistical difference, Kruskal‐Wallis test with a post hoc Dunn's test was used. (D–G) Kaplan–Meier plots of patients distinguished by RNA‐seq‐based EBV positivity in G1/G2 or G3 cases. (H, I) Kaplan–Meier plots of patients classified according to the number of EBV reads. Covariates were adjusted by IPW. [file FEB4-10-455-s008.pdf]

Figure S9

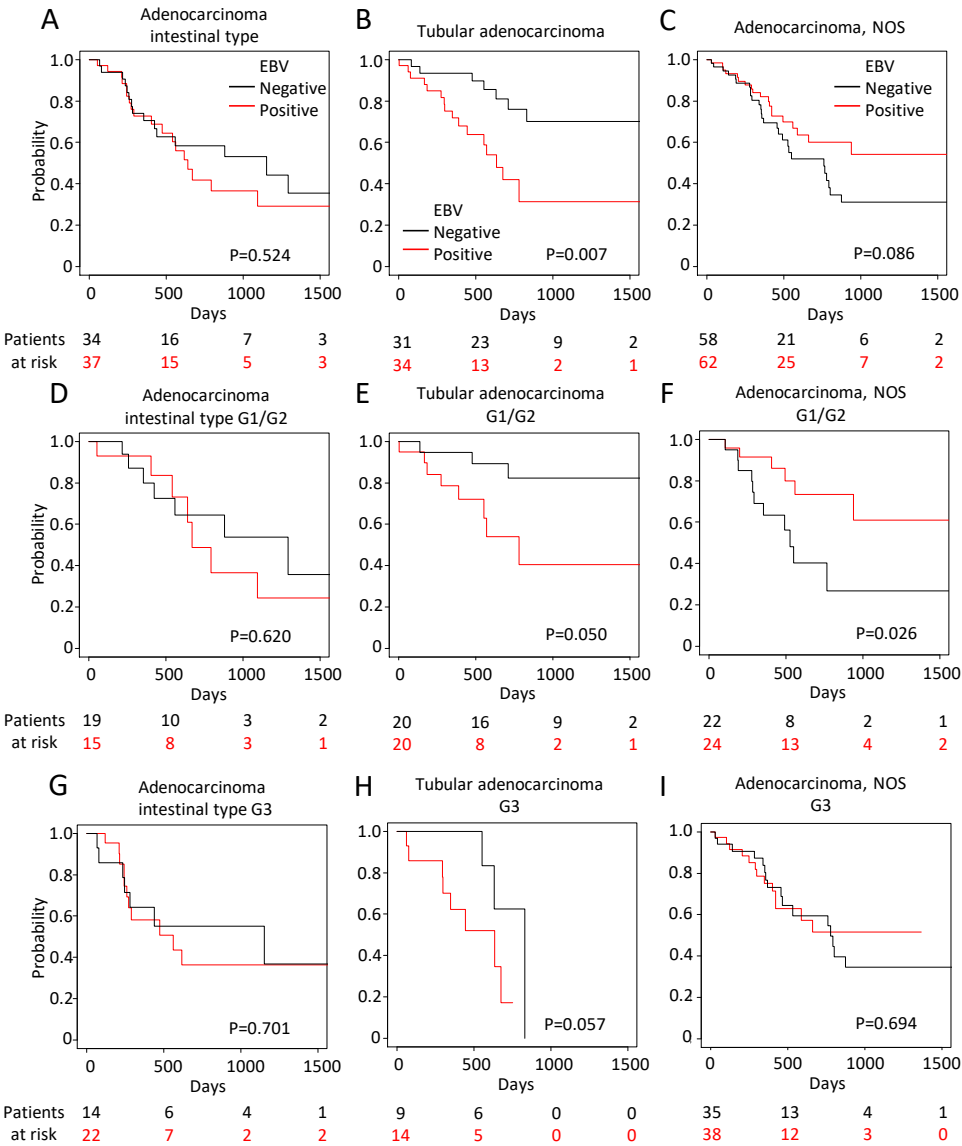

Supplement: Supplementary file 9 — Fig. S9. Unadjusted Kaplan–Meier curves corresponding to the data shown in Fig. 5. Prognostic effects of EBV‐positive and ‐negative case s classified according to tumor grade and the provisional histopathological categories. Intestinal type [adenocarcinoma intestinal type (A, D, G) and tubular adenocarcinoma (B, E, H)]. NOS type [adenocarcinoma NOS (C, F, I)]. [file FEB4-10-455-s009.pdf]

Figure S10

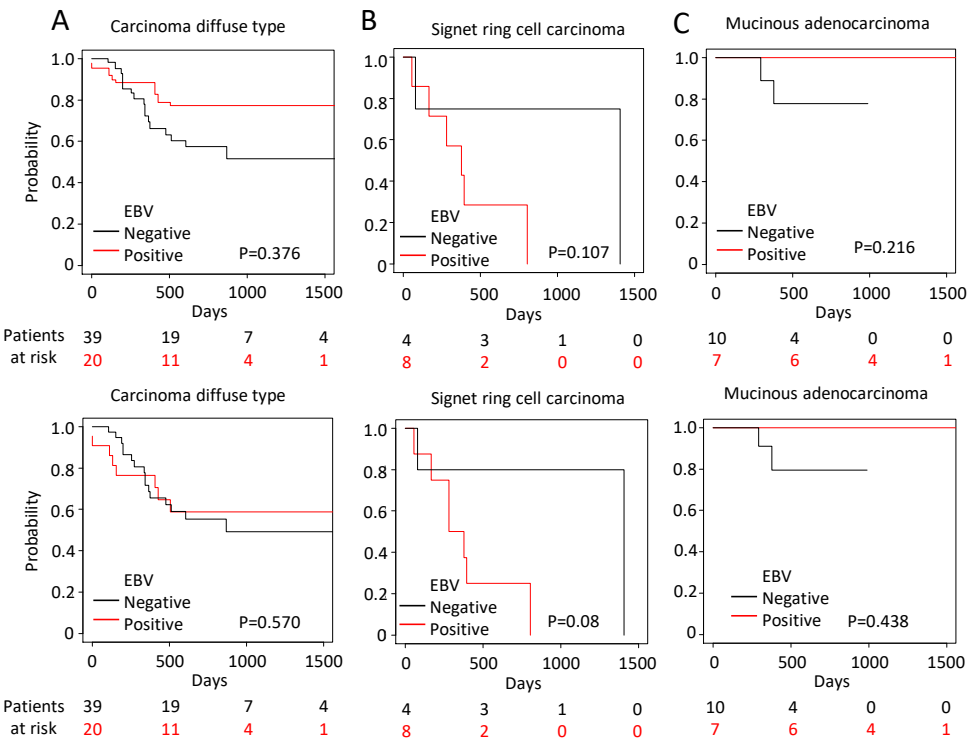

Supplement: Supplementary file 10 — Fig. S10. Kaplan–Meier curves of the remaining histopathological types (ICD‐O‐3‐harmonized) not listed in Fig. 5. (A) Carcinoma diffuse type, (B) signet ring cell carcinoma, and (C) mucinous adenocarcinoma with IPW‐adjustment (upper) or without IPW‐adjustment (lower). [file FEB4-10-455-s010.pdf]
