# Supplementary material for: Potential prognostic impact of EBV RNA‐seq reads in gastric cancer: a reanalysis of The Cancer Genome Atlas cohort
Source: FEBS Open Bio. 2020 Feb 16;10(3):455–67. doi: 10.1002/2211-5463.12803 (PMC7050242; doi:10.1002/2211-5463.12803)
Supplement: Supplementary file 11 — Table S1. Baseline characteristics of patients in the Cancer Genome Atlas‐Stomach Adenocarcinoma (TCGA‐STAD) dataset. [file FEB4-10-455-s011.pdf]

Table S1. Baseline Characteristics of Patients in the Cancer Genome Atlas-Stomach Adenocarcinoma (TCGA-STAD) dataset.

		No. cases Total n=375	%
Age	Mean (years-old)	66.43	
	Range (years-old)	35 - 90	
Sex	Male	241	64.26
	Female	134	35.73
Race	White	238	63.46
	asian	74	19.73
	black or african american	11	2.93
	native hawaiian or other pacific islander	1	0.26
	not reported	51	13.60
Tumor stage	stage i	2	0.53
	stage ia	14	3.73
	stage ib	37	9.86
	stage ii	27	7.20
	stage iia	35	9.33
	stage iib	49	13.06
	stage iii	3	0.80
	stage iiia	60	16.00
	stage iiib	52	13.86
	stage iiic	35	9.33
	stage iv	38	10.13
	not reported	23	6.13
subsite	Cardia	90	24.00
	Fundus	43	11.46
	Body	90	24.00
	Lesser curvature	1	0.26
	Antrum	134	35.73
	Pylorus	1	0.26
	not described	16	4.26
Primary diagnosis	Adenocarcinoma with mixed subtypes	1	0.26
	Adenocarcinoma, intestinal type	74	19.73
	Adenocarcinoma, NOS	137	36.53
	Carcinoma, diffuse type	61	16.26
	Mucinous adenocarcinoma	19	5.06
	Papillary adenocarcinoma, NOS	5	1.33
	Signet ring cell carcinoma	12	3.20
	Tubular adenocarcinoma	66	17.60
