# Supplementary material for: Potential prognostic impact of EBV RNA‐seq reads in gastric cancer: a reanalysis of The Cancer Genome Atlas cohort
Source: FEBS Open Bio. 2020 Feb 16;10(3):455–67. doi: 10.1002/2211-5463.12803 (PMC7050242; doi:10.1002/2211-5463.12803)
Supplement: Supplementary file 12 — Table S2. List of EBV DNA reference sequences for mapping next‐generation sequencing short reads. [file FEB4-10-455-s012.pdf]

Table S2. The List of DNA Reference Sequences of EBV to Map NGS Short Reads.

Gene names (EBV starain)	GenBank accession number
Human herpesvirus 4 strain Akata, complete genome	KC207813.1
Human herpesvirus 4 strain Mutu, complete genome	KC207814.1
Human herpesvirus 4 strain M81, complete genome	KF373730.1
Human herpesvirus 4 strain Raji, complete genome	KF717093.1
Human herpesvirus 4 DNA, complete genome, strain: 1 LGY-C666-1	AB828190.1
Human herpesvirus 4 DNA, complete genome, strain: 1 LGY-Raji	AB828191.1
Human herpesvirus 4 DNA, complete genome, strain: HN1	AB850643.1
Human herpesvirus 4 DNA, complete genome, strain: HN2	AB850644.1
Human herpesvirus 4 DNA, complete genome, strain: HN10	AB850645.1
Human herpesvirus 4 DNA, complete genome, strain: HN11	AB850646.1
Human herpesvirus 4 DNA, complete genome, strain: HN12	AB850647.1
Human herpesvirus 4 DNA, complete genome, strain: HN3	AB850648.1
Human herpesvirus 4 DNA, complete genome, strain: HN4	AB850649.1
Human herpesvirus 4 DNA, complete genome, strain: HN13	AB850650.1
Human herpesvirus 4 DNA, complete genome, strain: HN14	AB850651.1
Human herpesvirus 4 DNA, complete genome, strain: HN5	AB850652.1
Human herpesvirus 4 DNA, complete genome, strain: HN6	AB850653.1
Human herpesvirus 4 DNA, complete genome, strain: HN15	AB850654.1
Human herpesvirus 4 DNA, complete genome, strain: HN7	AB850655.1
Human herpesvirus 4 DNA, complete genome, strain: HN16	AB850656.1
Human herpesvirus 4 DNA, complete genome, strain: HN8	AB850657.1
Human herpesvirus 4 DNA, complete genome, strain: HN17	AB850658.1
Human herpesvirus 4 DNA, complete genome, strain: HN9	AB850659.1
Human herpesvirus 4 DNA, complete genome, strain: HN18	AB850660.1
Human herpesvirus 4 strain AG876, complete genome	DQ279927.1
Human herpesvirus 4 strain GD1, complete genome	AY961628.3
