# Supplementary material for: Potential prognostic impact of EBV RNA‐seq reads in gastric cancer: a reanalysis of The Cancer Genome Atlas cohort
Source: FEBS Open Bio. 2020 Feb 16;10(3):455–67. doi: 10.1002/2211-5463.12803 (PMC7050242; doi:10.1002/2211-5463.12803)
Supplement: Supplementary file 13 — Table S3. Provisional regrouped histopathological categories. [file FEB4-10-455-s013.pdf]

Table S3. Provisional regrouped histopathological categories.

Regrouped category	Primary Diagnosis (ICD-O-3-harmonized)
Diffuse Type	Carcinoma, diffuse type<br>Signet ring cell carcinoma
Intestinal Type	Adenocarcinoma, intestinal type<br>Tubular adenocarcinoma
Not otherwise specified (NOS) type	Adenocarcinoma, NOS<br>Papillary adenocarcinoma, NOS
Mixed Type	Mucinous adenocarcinoma<br>Adenocarcinoma with mixed subtypes
